# Graph-representation of oxidative folding pathways

**DOI:** 10.1186/1471-2105-6-19

**Published:** 2005-01-27

**Authors:** Vilmos Ágoston, Masa Cemazar, László Kaján, Sándor Pongor

**Affiliations:** 1 Bioinformatics Group, Biological Research Center, Hungarian Academy of Sciences, Temesvári krt. 62, 6726 Szeged, Hungary; 2 Protein Structure and Bioinformatics Group, International Centre for Genetic Engineering and Biotechnology, Area Science Park, 34012 Trieste, Italy; 3 Institute for Molecular Bioscience, University of Queensland, St. Lucia 4072, QLD, Australia

## Abstract

**Background:**

The process of oxidative folding combines the formation of native disulfide bond with conformational folding resulting in the native three-dimensional fold. Oxidative folding pathways can be described in terms of disulfide intermediate species (DIS) which can also be isolated and characterized. Each DIS corresponds to a family of folding states (conformations) that the given DIS can adopt in three dimensions.

**Results:**

The oxidative folding space can be represented as a network of DIS states interconnected by disulfide interchange reactions that can either create/abolish or rearrange disulfide bridges. We propose a simple 3D representation wherein the states having the same number of disulfide bridges are placed on separate planes. In this representation, the shuffling transitions are within the planes, and the redox edges connect adjacent planes. In a number of experimentally studied cases (bovine pancreatic trypsin inhibitor, insulin-like growth factor and epidermal growth factor), the observed intermediates appear as part of contiguous oxidative folding pathways.

**Conclusions:**

Such networks can be used to visualize folding pathways in terms of the experimentally observed intermediates. A simple visualization template written for the Tulip package  can be obtained from V.A.

## Background

The process of protein folding whereby a linear polypeptide chain reaches its native structure has been one of the most intensely studied biomolecular problems over the past 50 years (for current reviews see [1-3]). Folding of a protein is usually pictured as a search for the native conformation within the *conformational space *of all possible conformational states, each characterized by a set of parameters. Even though most of the conformational states are not accessible to experiment, graphic representations of the potential energy surface have played pivotal roles in explaining how the conformational space is gradually restricted during the process folding [4]. Key concepts such as folding pathways [5] are also best explained by graphic representations.

**Figure 1:**
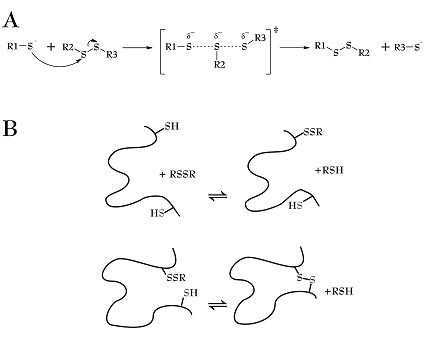
A. Thiol-disulfide exchange mechanism: in the pH range above 8, cysteine thiols are readily converted to thiolate anions (RS-), which are potent nucleophiles. RS- anions attack a disulfide bond, displacing one sulfur atom and forming a new bond with the other sulfur atom (nucleophilic substitution). The rate-determining step of this concerted process is the formation of a transition state with a partial transfer of the negative charge (δ^-^) over the three sulfur atoms. B. The formation of a disulfide bond on the polypeptide chain (solid curve) with the help of a small molecule reagent (thiol form: RSH, disulfide form: RSSR). The two steps both proceed via a thiol-disulfide exchange reaction. The first step shown is intermolecular and the second intramolecular. The rate of the intramolecular step is relevant to protein folding, since it also involves conformational changes.

The particular kind of folding that this article is concerned with is oxidative folding, which is the fusion of native disulfide bond formation with conformational folding [6-8]. This complex process is guided by two types of interactions: first, non-covalent interactions giving rise to secondary and tertiary structure, and second, covalent interactions between cysteine residues, which ultimately transform into native disulfide bridges. The process of disulfide formation is a simple chemical reaction in which two SH groups join to form a disulfide link (Figure 1A). If the SH groups are on a polypeptide chain, the *in vitro *reaction can be promoted by an external redox system such as a mixture of oxidized and reduced glutathione, or cysteine and cystine, respectively. *In vivo*, the oxidative power comes from specific agents such as the molecular chaperones protein disulfide isomerases[9].

The underlying chemical mechanism is disulfide interchange (Figure 1B). In this scheme there are two kinds of reactions: i) in a *redox reaction *a protein disulfide bond is created (or abolished), i.e. the oxidative state of the polypeptide is changed. This is the case when one of the participants of the reaction (say RSH) is not part of the protein. ii) In a *shuffling reaction *both participants of the reaction are protein-bound, so the oxidative state of the polypeptide does not change. In view of these possibilities it becomes obvious that there are a great many ways in which disulfide bridges can form and rearrange during the folding process. Today it is generally accepted that non-covalent interactions guide the process of folding and formation of disulfide bridges will lock the protein into the right conformation. The advantage of oxidative folding as opposed to general protein folding is that disulfide intermediates can be chemically isolated and studied using such techniques as acid trapping of the intermediates and analysis of the disulfide bridges using a combination of enzymatic cleavage and mass spectrometry. There is a body of literature in describing the pathways of oxidative folding in terms of disulfide intermediates [6-8], and our goal is show how graph theory can be used to visualize them.

Graph theory has been applied to many aspects of protein research (for a recent review see [10]). The applications followed two broad avenues:

i) First, protein structure itself can be considered as a graph consisting of various interactions (such as covalent bonds, hydrogen bonds, spatial vicinities, contacts etc.) as edges, the nodes being atoms or residues of the protein. For instance, one of the classical definitions of protein secondary structures is based on main/chain H-bond contacts between residues [11]. Structural neworks have been used in folding research as well. It was found, among others, that the so-called contact order, i.e. the average sequence distance between residues in atomic contact, seems to be a key determinant of folding speed [12]. Another line of research concentrates on characteristic networks of inter-atomic contacts that may form stabilization centres in protein structures and can be the reason of the differential stability of various proteins [13,14]. It was found that populated conformations seen in molecular dynamics simulations contain characteristic networks of residues [15,16].

ii) In the network descriptions of the folding space, on the other hand, the folding states are the nodes, and transitions are the edges between them. This approach was fostered by the finding that the robustness and stability of networks may be the result of simple topological properties that are invariant throughout various technical as well as biological systems [17]. In the following years the network topology of a large number of systems have been described, and it was found that some topology classes, such as those characterized by a scale-free distribution of the number of links at each node, or the so called "small world models" that are characterized by densely connected subnetworks loosely linked between each other, are indeed found in various systems within and without biology (for reviews see [18]). The various network types were described in terms of simple measures borrowed from graph theory, such as the clustering coefficient, the diameter of the graph etc [19]. Scala and associates described the folding states of short peptides using Monte Carlo simulation on lattice models [20]. They found that that the geometric properties of this network are similar to those of small-world networks, i.e. the diameter of the conformation space increases for large networks as the logarithm of the number of conformations, while locally the network appears to have low dimensionality. Shakhnovich and co-workers analysed the folding states of proteins during molecular dynamics simulations. It was found that the folding space is reminiscent of scale-free network, characterized by a majority of less populated states as well as some highly populated states reminiscent of "hubs" seen in other systems [21].

Our purpose is to describe the folding space of the oxidative folding process using graph representations. This is an intriguing task since, in contrast to "ordinary" protein folding, the number of states defined in terms of disulfide links is not exceedingly high, moreover the actual disulfide intermediates can be isolated and studied. We will approach the problem in two steps as: i) We will use graph theory to describe the disulfide intermediates, and to enumerate the states of the folding space. ii) Then we will represent the folding space as a network (graph) of all possible intermediates. We show with few examples that experimentally observed intermediates mapped onto this network appear as contiguous folding pathways.

## Results and discussion

### Graph representation of oxidative folding intermediates

**Figure 2:**
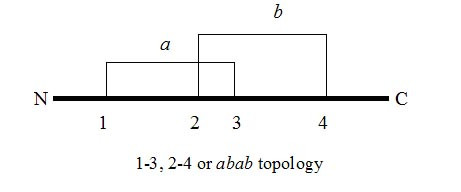
Nomenclature for disulfide topologies. Disulfides can be labeled by the sequence positions, or simply by the sequential number of the cysteine residues they connect (*1–3*, *2–4 *topology). Alternatively, it is customary to alphabetically label the disulfide bridges, and and to assign bridge label to the cysteines, starting from the N terminus (*abab *topology).

The disulfide topology of a protein is unequivocally defined by describing which cysteines are connected to each other. For example, a topology "1–3, 2–4" means that a protein with four cysteines has two disulfide bridges that connect cysteines (1,3) and cysteines (2,4) respectively. Cysteines can be labelled by their sequence position, or – as in the previous example – in a serial order from the N-terminus (Figure 2).

The number of fully oxidised (disulfide bonded) isomers in a protein chain with *n *disulfide bonds (2*n *cysteines) can be deduced from simple combinatorial considerations as (2*n*)!/(*n*!*2^*n*^). According to this formula proteins with two disulfide bridges have 3 fully oxidized isomers, 3-disulfide proteins have 15 and 4-disulfide proteins have 105. In other words, the number of intermediates increases very fast as a function of the number of constituent cysteines.

**Figure 3:**
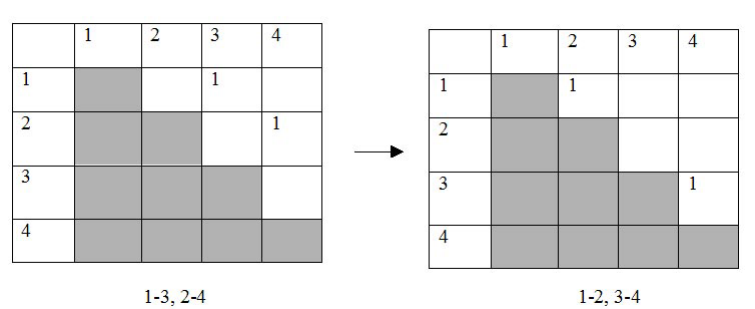
Adjacency matrices of two disulfide topologies of a peptide with two disulfide bridges

For a complete description of the folding process we have to consider both fully oxidized intermediates as well as the ones with free cysteine residues. For this purpose we will use a formal description of the intermediates as undirected graphs, with cysteines as nodes and disulfide bridges as edges (the main chain will not be represented). For the majority of naturally occurring protein structures the resulting graphs will be extremely simple. If the protein has *n *cysteines, the *n *× *n *adjacency matrix of the graph is symmetrical; it will contain a value of 1 if two cysteines form a disulfide bond and zero otherwise. As one cysteine can form only one disufide bridge, each column and each row will have at most one value of 1. Examples are shown in Figure 3.

#### Description of the oxidative folding space as graphs

The transitions between folding intermediates can be conveniently described by comparing the adjacency matrices of the two states. For the enumeration of the transition reactions we introduce a few simple variables. *NB *is the number of disulfide bonds, calculated as the sum of the elements of the adjacency matrix.


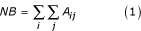


The sum of elements in the i-th column plus the i-th row,


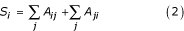


is 1 if the i-th cysteine is part of a disulfide bridge and zero otherwise. The sum of the differences calculated between the *S*_*i *_measures of two adjacency matrices,


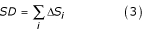


shows how many cysteines gain or loose a bond as the molecule passes from one state to the other. Here we are interested only in the two kinds of elementary reactions depicted in Figure 1B. In *shuffling reactions*, the number of disulfide bridges *NB *remains the same by definition, and it is easy to show that *SD *will differ exactly by 2. In *redox steps *in which one disulfide bridge is established or lost, *NB *and *SD *will increase or decrease by one and two, respectively.

On the above basis one can easily draw a network of all possible oxidative folding pathways. For a protein of *n *cysteines, we first generate the graphs (adjacency matrices) of all possible intermediates, i.e. those with 0,1...(i ≤ n/2) disulfide bridges. Then we compare all pairs of intermediates in terms of the above parameters. A shuffling edge will be drawn between two intermediate states if |SD| = 2 and ΔNB = 0; redox edges will be drawn if |SD| = 2 and ΔNB = 1. If the values of |SD| and ΔNB are different from these two combinations, no edge will be drawn.

The graph characteristics of a few systems are summarized in Table 1. The results show that as the number of cysteines increases, the clustering coefficient of the system decreases. While the average path length increases. Both findings are consistent with the intuitive view that the folding space of peptides with many cysteines may be too complex and thus the systems may be unable to fold fast enough.

**Figure 4:**
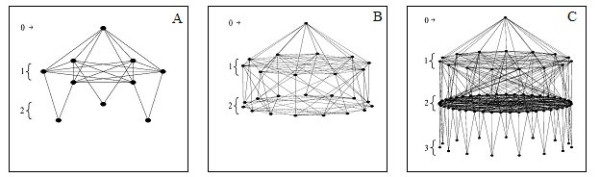
Three dimensional representation of the oxidative folding space of polypeptides with 4, 5 and 6 cysteine residues (A, B and C, respectively). The nodes represent intermediates, the number of disulfide bridges is indicated with numbers on the left of each panel. The edges indicate disulfide exchange transitions. Zero indicates the fully reduced state, nodes in the lowest plane are the fully oxidized intermediates, one of which is usually the native state. Edges within the same plane indicate shuffling reactions (interchange between two protein-bound disulfides), edges between planes are redox transitions in which a disulfide bridge is created or abolished. A simple visualization tool written for the Tulip package  can be obtained from V.A. vilagos@brc.hu

The pathways can also be graphically represented, and in order to simplify the resulting picture, we chose a 3D representation wherein the states (species) are grouped according to the number of disulfide bridges (Figure 4). Species with the same number of disulfide bridges are placed on the same plane, so shuffling reactions, which do not change the number of disulfide bridges are represented as edges within the same plane. It is noted that on each of the separate planes we find a regular graph. This is not surprising: exhaustive enumeration of theoretical states, such as Eigen's quasi-species [22], can produce highly connected regular graphs. On the contrary, reactions in which a disulfide bridge is gained or lost, are represented as edges between two neighbouring planes. The fully reduced state (zero disulfide bridges) is on top, the fully oxidized species, one of which is the native state, is on the bottom.

Panel B shows a peptide with an odd number cysteines, such as granulocyte-colony stimulating-factor [23,24] in which the native state contains one free cysteine residue. In this case there are shuffling edges even in the lowest plane in the figure, so the native state (one of the states in the lowest plane) can be subject to shuffling transitions. On the contrary, if the number of cysteines is an even number (i.e. in the majority of known proteins), the fully oxidized DISs can not interconvert into each other in one step. In some cases however an additional cysteine is in fact used to facilitate the process of oxidative folding: the propeptide of BPTI contains an additional free cysteine that seems to significantly speed up the *in vitro *folding of the molecule [25]. *In vivo*, the propeptide is subsequently cleaved, and in this way the structure is locked into the native disulfide configuration.

**Figure 5:**
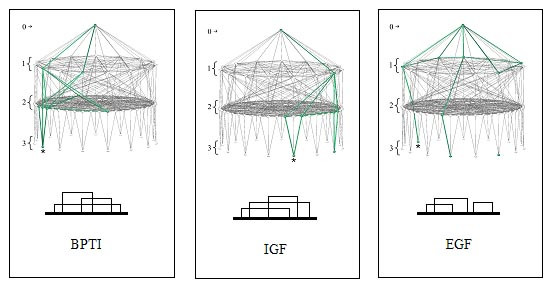
The oxidative folding pathways of bovine pancreatic trypsin inhibitor (BPTI), insulin-like growth factor (IGF) and epidermal growth factor (EGF). The disulfide connectivity of the intermediates is sumarized in Table 2. Asterisk denotes the native state.

In principle, the oxidative folding pathways can be pictured as routes within the full network, starting at the fully reduced species and ending at the native state. In the literature there are a few well-studied examples in which folding intermediates have been determined. The experimentally observed disulfide intermediates of three examples, bovine pancreatic trypsin inhibitor, insulin-like growth factor and epidermal growth factor, are shown in Table 2 and Figure 5, respectively. It is noted that experimental methods do not necessarily reveal all possible intermediates; some of the intermediates may be too short-lived or not abundant enough so as to be noticed an isolated. In spite of these limitations, the folding pathways appear as connected subgraphs within the network of all possible intermediates, showing that the experimental techniques actually identified states that can interconvert into one another. Only in EGF do we see an "isolated" intermediate, which suggests that some intermediates of the pathway were not observed experimentally.

## Conclusions

The oxidative folding space of polypeptides can be represented as networks in which the nodes are the disulfide intermediates while the edges are transitions between them. A simple visualization tool written was developed to draw 3D pictures of such networks in which the states having the same number of disulfide bridges are placed on separate planes. These pictures provide a simple method for the visualization of oxidative folding pathways as studied by experimental methods. In the case of bovine pancreatic trypsin inhibitor, insulin-like growth factor and epidermal growth factor, the folding pathways appear as a small network of contiguous routes that connect the fully reduced state to the native state. A further plausible extension of this method would include colouring of the folding states by quantitative properties and look for correlations between the coloured areas of the network and the experimentally determined folding pathways.

Even though the topology of the theoretically complete folding space appears to be highly regular, data currently available are insufficient to draw general conclusions on the topology of the experimentally observed folding pathways. Experimentalists find folding intermediates as a series of chromatographic peaks, and usually the disulfide bridges of more abundant species are analysed first. The question whether or not all the relevant intermediates have been analyzed is difficult to answer, and mapping the intermediates onto the graphs presented here may help one to decide whether or not the pathways found are contiguous.

## Authors' contributions

V.A. designed and implemented the algorithms and carried out the calculations. M.C. helped to compile the experimental data and to draft the manuscript. L.K. designed the representation of folding intermediates. The project was coordinated by S.P.

## Figures and Tables

**Table 1:** Parameters of oxidative folding networks*

N of cysteines	N of intermediates (nodes)	Redox transitions	Shuffling transitions	Total no of transitions (edges)	Clustering coefficient C	Average path length
1	1	0	0	0	1.000	0.000
2	2	1	0	1	1.000	1.000
3	4	3	3	6	1.000	1.000
4	10	12	12	24	0.400	1.467
5	26	40	60	100	0.410	1.810
6	76	150	240	390	0.247	2.293
7	232	546	1050	1596	0.253	2.640
8	764	2128	8736	10864	0.181	3.149
9	2620	8352	19152	27504	0.182	3.550
10	9496	34380	83520	117900	0.142	3.977

*Oxidative folding networks represent the entire folding space that comprise all possible intermediates. Folding pathways, on the other hand, include only the experimentally observed intermediates.

**Table 2:** Disulfied intermediates experimentally observed in the oxidative folding of various proteins

Protein	Disulfide intermediates^1^	Ref.
Bovine pancreatic trypsin inhibitor (BPTI)	3–5; 1–6; 3–5, 1–2; 3–5, 1–4; 3–5, 2–4; 1–6, 2–4; 3–5, 1–6; **1–6, 3–5, 2–4**;	[26, 27]

Insulin-like growth factor (IGF)	2–6; 2–6, 3–5; 2–6, 1–4; 2–6, 4–5; 2–6, 1–3; 2–6, 1–3, 4–5; **1–4, 2–6, 3–5;**	[28-30]

Epidermal growth factor (EGF)	2–3; 1–2; 4–6; 5–6; 3–4; 2–4, 5–6; 2–5, 3–4; 1–6, 2–5, 3–4; 1–2, 3–4, 5–6; **1–3, 2–4, 5–6;**	[31]

^1^The intermediates are described with the notation given in Figure 2. The native disulfide connectivity is in bold, the fully reduced species is not included.
